# Evaluating the Outcomes of Surgical Hip Dislocation for Femoral Head Trauma: A Systematic Review

**DOI:** 10.7759/cureus.97152

**Published:** 2025-11-18

**Authors:** Muaz Hassan, Hisham A. A. Elnosh, Elrazi Nagemeldin Khidir Mohamed, Yusra Ahmed Mohamedzein Adam, Ashraf Eltag Elamin Ahmed, Dalia Mohamedalamin Mohamedalhassan Abbas, Omer Kamal Ahmed Omer

**Affiliations:** 1 Department of Orthopaedics, Wrexham Maelor Hospital, Wrexham, GBR; 2 Microbiology, Immunology and Pharmacology, St. George's University, True Blue, GRD; 3 General Surgery, Dr. Abdul Rahman Al Mishari Hospital, Alolaya, SAU; 4 Physiology, St. George's University, True Blue, GRD; 5 Orthopaedics, Armed Hospitals Southern Region, Khamis Mushayt, SAU; 6 General Surgery, King Salman Bin Abdulaziz Medical City, Madina, SAU; 7 Trauma and Orthopaedics, Luton and Dunstable Hospital, Bedfordshire, GBR

**Keywords:** avascular necrosis, femoral head fracture, ganz approach, orthopaedic trauma, pipkin classification, surgical hip dislocation, systematic review, trochanteric flip osteotomy

## Abstract

Femoral head fractures are complex injuries often associated with high-energy trauma and hip dislocation. Surgical hip dislocation (SHD), particularly the Ganz trochanteric flip osteotomy, was developed to provide extensive intra-articular access while preserving the femoral head's vascular supply. However, a consolidated synthesis of its outcomes is needed. This systematic review aims to evaluate the clinical and radiological outcomes, complication profiles, and key technical considerations of SHD for the management of femoral head trauma.

A systematic literature search was conducted in accordance with the Preferred Reporting Items for Systematic Reviews and Meta-Analyses (PRISMA) 2020 guidelines across five databases (PubMed, Scopus, Embase, Web of Science, and Cochrane Library) on October 2, 2025. Studies reporting functional or radiological outcomes of SHD for femoral head trauma were included. The risk of bias was assessed using the ROBINS-I tool. Data on study characteristics, functional scores, and complications were extracted and synthesized narratively due to heterogeneity.

Ten studies involving 267 patients were included. The majority of studies reported good to excellent functional outcomes, with mean modified Harris Hip Scores and Merle d'Aubigné scores indicating satisfactory recovery. The rate of avascular necrosis (AVN) was consistently low to moderate, ranging from 0% to 8.3% in most studies, a rate notably lower than historically reported for traditional posterior approaches. However, Pipkin type IV fractures were associated with a poor prognosis, with one study reporting an 87.5% rate of post-traumatic arthritis and a 57.1% conversion to total hip arthroplasty. Heterotopic ossification was a common but often asymptomatic finding. The comparative analysis underscored that early hip reduction (less than six hours) significantly improved outcomes. The overall risk of bias was low for all included prospective and retrospective cohort studies, while the two case series were appropriately judged to have a serious risk of bias inherent to their design.

SHD is a safe and effective approach for femoral head trauma, facilitating anatomical reduction and yielding good functional outcomes with a low risk of AVN. The Ganz trochanteric flip osteotomy is the cornerstone technique. Prognosis is highly dependent on fracture type, with Pipkin IV injuries posing the greatest challenge, and the timing of surgical intervention is a critical modifiable factor for success.

## Introduction and background

Femoral head fractures are uncommon but serious injuries that often occur in association with high-energy trauma, such as motor vehicle accidents or falls from significant heights [1]. These injuries are frequently accompanied by posterior hip dislocations, acetabular fractures, and damage to the surrounding soft tissue structures, posing considerable challenges in achieving both anatomic reduction and preservation of the femoral head’s vascular supply [2]. Owing to their complexity, the optimal surgical approach for managing femoral head trauma remains a subject of ongoing debate among orthopedic surgeons.

Surgical hip dislocation (SHD), first systematically described by Ganz et al. [3] in 2001, has emerged as a versatile and safe technique that allows full visualization of the femoral head and acetabulum while minimizing the risk of compromising the blood supply to the femoral head. This approach provides 360° exposure of the femoral head, facilitating accurate reduction, fixation, and management of associated intra-articular pathologies [4]. Consequently, SHD has been increasingly adopted for the treatment of femoral head fractures, acetabular injuries, and certain cases of femoroacetabular impingement [5].

Despite its theoretical and technical advantages, the outcomes of SHD for femoral head trauma remain inconsistently reported across the literature. Studies have demonstrated variable results in terms of functional recovery, radiological healing, and complication rates-including avascular necrosis (AVN), post-traumatic arthritis, and heterotopic ossification [6]. These discrepancies may arise from differences in injury patterns, timing of intervention, surgical expertise, and postoperative rehabilitation protocols [7]. Furthermore, most available studies are limited by small sample sizes and heterogeneous methodologies, making it difficult to draw definitive conclusions regarding the efficacy and safety of SHD in this context.

Given these uncertainties, a comprehensive synthesis of the existing evidence is warranted. This systematic review aims to critically evaluate and summarize the clinical and radiological outcomes of surgical hip dislocation in the management of femoral head trauma. By consolidating findings from recent original studies, this review seeks to clarify the procedure’s effectiveness, identify common complications, and highlight areas where further research is needed to optimize patient outcomes and guide clinical decision-making.

## Review

Methods

Review Design and Protocol

This systematic review was conducted in accordance with the Preferred Reporting Items for Systematic Reviews and Meta-Analyses (PRISMA) 2020 guidelines [8] to ensure methodological rigor and transparency. The review protocol was designed prior to the initiation of the literature search to establish clear eligibility criteria, data extraction methods, and risk of bias assessment procedures.

Eligibility Criteria

The inclusion and exclusion criteria were developed using the Population, Intervention, Comparison, Outcomes, Study design (PICOS) framework. Studies were included if they investigated clinical or radiological outcomes following SHD for femoral head trauma. Only original studies involving human subjects were considered eligible. The inclusion criteria were as follows: (1) patients diagnosed with femoral head fractures or associated injuries treated using SHD, (2) studies reporting postoperative clinical or radiological outcomes, and (3) studies published in English between January 01, 2015, and October 02, 2025. Exclusion criteria included non-human studies, case reports, reviews, editorials, technical notes, and studies with incomplete or non-extractable data (Table 1).

**Table 1:** Eligibility criteria based on Population, Intervention, Comparison, Outcomes, Study design (PICOS) framework AVN, avascular necrosis

Parameter	Description
Population (P)	Patients with femoral head pathology requiring direct intra-articular access, including femoral head fractures or osteonecrosis.
Intervention (I)	Surgical Hip Dislocation (SHD).
Comparison (C)	Other surgical approaches or absence of comparison (single-arm studies were also included).
Outcomes (O)	Functional outcomes (e.g., Harris Hip Score), radiological results, and postoperative complications such as AVN, heterotopic ossification, and post-traumatic arthritis.
Study Design (S)	Original studies including retrospective and prospective cohort studies, and comparative studies.

Information Sources

A comprehensive literature search was conducted across the following electronic databases: PubMed, Scopus, Embase, Web of Science, and the Cochrane Library. The search covered studies published from January 01, 2015, and October 02, 2025. To ensure completeness, citation searching of relevant reviews and included studies was performed to identify any additional eligible studies that might not have been captured in the initial database search.

Search Strategy

The search strategy combined Medical Subject Headings (MeSH) and free-text terms related to “surgical hip dislocation,” “femoral head fracture,” “femoral head trauma,” and “outcomes.” Boolean operators (AND, OR) were used to refine the search. The complete search strategy was adapted for each database to account for differences in indexing.

Selection Process

All identified records were imported into EndNote X9 (Clarivate Analytics) for reference management. Duplicate entries were automatically and manually removed. Two independent reviewers screened titles and abstracts for relevance. The full texts of potentially eligible studies were then retrieved and evaluated against the inclusion criteria. Any disagreements between the reviewers were resolved through discussion or consultation with a third reviewer.

Data Collection Process

Data extraction was performed independently by two reviewers using a standardized data extraction form. Extracted data included study characteristics (authors, year, country, study design, and sample size), patient demographics (age, gender), type of injury, surgical details, duration of follow-up, and clinical and radiological outcomes. Discrepancies were resolved through discussion to ensure accuracy and consistency.

Data Items

Key data items collected included mean postoperative functional scores (e.g., Harris Hip Score), rates of avascular necrosis (AVN), incidence of heterotopic ossification, and occurrence of post-traumatic arthritis. Additionally, information on complications, reoperations, and patient satisfaction was recorded when available.

Study Risk of Bias Assessment

The methodological quality and risk of bias of included studies were assessed using the Risk Of Bias In Non-randomized Studies - of Interventions tool (ROBINS-I) [9]. This tool evaluates potential biases across seven domains, including confounding, participant selection, intervention classification, deviations from intended interventions, missing data, outcome measurement, and selective reporting. Each study was rated as having low, moderate, serious, or critical risk of bias in each domain.

Effect Measures

Where applicable, continuous outcomes were expressed as mean ± standard deviation (SD), and categorical outcomes as percentages. However, due to significant heterogeneity among studies in terms of outcome measures, study designs, and follow-up durations, a meta-analysis was not performed. The data were instead synthesized narratively.

Synthesis Methods

Given the wide variability in reporting standards, patient populations, and definitions of outcomes across the included studies, a quantitative synthesis was deemed inappropriate. Specifically, differences in scoring systems (e.g., Harris Hip Score vs. Merle d’Aubigné Score), variations in surgical technique, and inconsistent reporting of complications precluded meaningful statistical pooling. Therefore, a qualitative synthesis was performed, summarizing the results descriptively and identifying common trends and patterns.

Results

Study Selection Process

**Figure 1:**
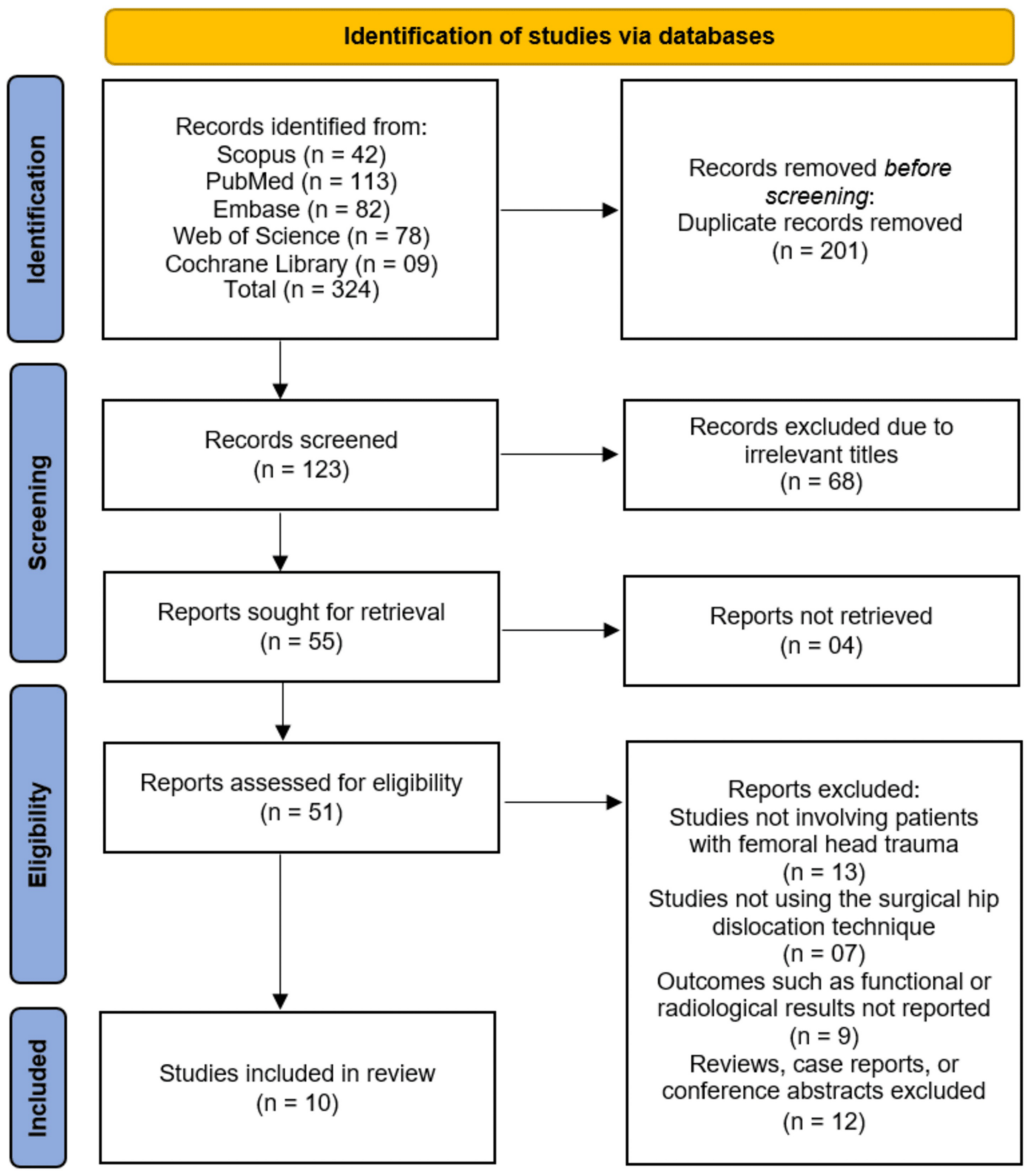
Preferred Reporting Items for Systematic Reviews and Meta-Analyses (PRISMA) Flow Diagram of Studies Selection Process

The systematic search across five databases (Scopus, PubMed, Embase, Web of Science, and Cochrane Library) initially identified 324 records. After the removal of 201 duplicate records, 123 unique studies were screened based on their titles and abstracts. This screening process led to the exclusion of 68 records that were deemed irrelevant. The full texts of the remaining 55 articles were sought for retrieval, of which four could not be obtained. The 51 successfully retrieved reports were then assessed for eligibility in detail. Of these, 41 studies were excluded for the following reasons: 13 did not involve patients with femoral head trauma, seven did not utilize the surgical hip dislocation technique, nine did not report the relevant functional or radiological outcomes, and 12 were reviews, case reports, or conference abstracts. Ultimately, this rigorous selection process resulted in 10 studies that met all the inclusion criteria and were incorporated into the systematic review [10-19] (Figure 1).

Study Characteristics

A total of 10 studies [10-19] involving 267 patients were included in this systematic review to evaluate the outcomes of SHD for femoral head trauma. The characteristics of these studies are summarized in Table 2. The included studies were published between 2015 and 2024 and originated from a diverse range of countries, including China [10,11,17], Egypt [13,14], Italy [12,19], Korea [13], India [18], and the USA [16]. All studies were either retrospective in design [10,11,13,14,16,19] or prospective cohorts [15,17,18], with one study being a case series [12]. The sample sizes varied, ranging from five [12] to 71 [11] patients, with a mean age of patients typically in the third to fourth decade of life.

**Table 2:** Characteristics of Included Studies M/F, Male/Female; NR, Not Reported; ONFH, Osteonecrosis of the Femoral Head; SD, Standard Deviation; SHD, Surgical Hip Dislocation; THA, Total Hip Arthroplasty; TFO, Trochanteric Flip Osteotomy; 3D, Three-Dimensional.

Author (Year)	Country	Study Design	Sample Size (n)	Mean Age (years)	Gender (M/F)	Type of Femoral Head Injury (Pipkin Classification)	Surgical Approach	Follow-up Duration
Liang et al., [10] (2024)	China	Retrospective study	39	35.1	28 males and 11 females	Non-traumatic osteonecrosis of the femoral head	Surgical hip dislocation through femoral head fovea fenestration and impaction bone grafting	24–72 months (mean 60 ± 13.0 months)
Wang et al., [11] (2021)	China	Retrospective comparative study	71	42.6 and 39.2	24/15 and 24/8	Femoral head fracture with posterior hip dislocation	Ssurgical treatment after hip reduction	66.3 ± 8.4
De Mauro et al., [12] (2021)	Italy (Rome)	Case series	5	39.8	4/1	Pipkin fractures	Gibson approach + Ganz trochanteric flip osteotomy	24 months (range 12–24)
Yoon et al., [13] (2022)	Korea	Retrospective	34	37.9	29/5	II: 7, III: 2, IV: 25	Trochanteric Flip Osteotomy with Surgical Hip Dislocation (TFO with SHD)	5.1 years (range 2.8–10.5)
Hosny et al., [14] (2022)	Egypt	Retrospective	18	32	12/6	Pipkin I: 6, Pipkin II: 12	Surgical hip dislocation (Ganz technique)	≥24 months
Khalifa et al., [15] (2021)	Egypt	Prospective cohort	31 (27 at follow-up)	33.8	NR	NR	SHD	48 months (mean)
Engel et al., [16] (2021)	USA	Retrospective chart review	10	39.57	NR	Pipkin IV	Kocher–Langenbeck approach with trochanteric flip (6/7 patients); fragment excision (1 patient)	NR (mean THA conversion at 20.5 months reported for some)
Wang et al., [17] (2019)	China	Prospective study	17 (12 completed follow-up)	39.9 ± 12.2	8/4	Type I: 4, Type II: 3, Type IV: 5	3D printing-based Ganz approach	Mean 35 months (range 25–48)
Gavaskar and Tummala, [18] (2015)	India	Prospective, non-randomised	28	NR	NR	Type I/II Pipkin fracture dislocation	Ganz approach (safe surgical dislocation)	Mean 36 months
Massè et al., [19] (2015)	Italy	Retrospective Case Series	13	<55 (exact mean not specified)	NR	Displaced femoral head fractures (includes Pipkin IV for associated acetabular fractures)	Surgical hip dislocation via digastric trochanteric flip osteotomy	Minimum 24 months (mean 77 months, SD 32.8 months)

The primary indication for SHD was the management of femoral head fractures, with the majority of studies focusing on Pipkin-type fractures [12-14,16-19]. One study investigated the application of a similar technique for non-traumatic osteonecrosis of the femoral head (ONFH) [10]. The surgical approach was predominantly the Ganz trochanteric flip osteotomy with surgical hip dislocation, which was utilized in the majority of studies [12-15,17-19]. Other approaches included the Gibson approach combined with the Ganz technique [12], the Kocher-Langenbeck approach with a trochanteric flip [16], and a unique technique involving femoral head fovea fenestration with impaction bone grafting [10]. One study utilized a 3D printing-based Ganz approach for preoperative planning [17]. The mean follow-up duration across the studies was generally substantial, ranging from 24 months to a mean of 77 months, allowing for the assessment of medium- to long-term outcomes [19].

Functional Outcomes

Postoperative functional outcomes were assessed using various validated hip scoring systems. As detailed in Table 3, several studies reported good to excellent functional results. For instance, De Mauro et al. [12] reported a mean modified Harris Hip Score (mHHS) of 92.1, with four out of five patients achieving an excellent outcome on the Merle d'Aubigné score. Similarly, Gavaskar and Tummala [18] reported a mean Modified Merle d'Aubigné score of 16.5 (range 14-18), indicating good to excellent function. Massè et al. [19] also documented satisfactory outcomes with a mean Modified Harris Hip Score of 82. In a study utilizing a 3D printing-assisted technique, Wang et al. [17] reported that six out of 12 patients had an excellent result and four had a good result according to the Modified Merle d'Aubigné score. These findings collectively suggest that SHD facilitates anatomical reduction and stable fixation, leading to favorable functional recovery in most patients.

**Table 3:** Clinical and Radiological Outcomes of Surgical Hip Dislocation AVN, Avascular Necrosis; HHS, Harris Hip Score; HO, Heterotopic Ossification; MCID, Minimal Clinically Important Difference; mHHS, modified Harris Hip Score; NR, Not Reported; ONFH, Osteonecrosis of the Femoral Head; SD, Standard Deviation; SF-12, Short Form-12 Health Survey; THA, Total Hip Arthroplasty; VAS, Visual Analog Scale; WOMAC, Western Ontario and McMaster Universities Osteoarthritis Index.

Author (Year)	Functional Outcome (Score Used)	Mean Score ± SD	Rate of Avascular Necrosis (AVN)	Post-traumatic Arthritis (%)	Heterotopic Ossification (%)	Reoperation Rate (%)	Main Conclusion
Liang et al., [10] (2024)	HHS & MCID	NR	NR	NR	2.6	30.8	SHD via fovea fenestration and bone grafting is safe and effective for non-traumatic ONFH; early weight-bearing increases failure risk.
Wang et al., [11] (2021)	Thompson-Epstein, Merle D’Aubigné & Postel, VAS, SF-12	NR	Higher in delayed	Higher in delayed	NR	NR	Early hip reduction (<6h) improves healing, function, and reduces complications vs delayed (6–12h)
De Mauro et al., [12] (2021)	mHHS, Vail, WOMAC (Pain, Stiffness, Function), Merle d’Aubignè	mHHS 92.1 (75.9–100), Vail 81.8 (55–95), WOMAC Pain 1.4 (0–7), Stiffness 1.2 (0–6), Function 6.4 (0–22), Merle d’Aubignè 4 excellent / 1 good	0%	NR	0%	0%	Gibson + Ganz approach allows anatomical reduction of femoral head fractures with good short-term clinical and radiological outcomes.
Yoon et al., [13] (2022)	Merle d'Aubigné–Postel / Thompson–Epstein	14.4 (range 8–17) / 22 patients with good/excellent	2/34 (5.9%)	NR	NR	NR	TFO with SHD is a safe and useful approach for FHFD; high AVN risk when associated with displaced femoral neck fractures
Hosny et al., [14] (2022)	Harris Hip Score / Modified Merle d’Aubigne & Postel	NR	5.6% (1/18)	NR	NR	0%	Surgical hip dislocation via Ganz approach provides satisfactory functional and radiological outcomes with low complication rate.
Khalifa et al., [15] (2021)	Modified Harris Hip & Modified Merle d’Aubigne and Postel Scores	NR	2/27 (7.4%)	1/27 (3.6%)	5/27 (18.5%)	0%	SHD is safe and effective for femoral head fractures, allowing anatomical reduction with acceptable complication rates.
Engel et al., [16] (2021)	NR	NR	NR	87.5%	NR	57.1% (conversion to THA)	High rates of osteonecrosis and post-traumatic arthritis; worse than previously reported, possibly due to longer follow-up and associated comminuted fractures
Wang et al., [17] (2019)	Modified Merle d’Aubigne score	6 excellent, 4 good, 2 poor	1/12 (8.3%)	NR	4/12 (33.3%)	1/12 (8.3%)	Safe, effective, preserves blood supply, good hip function, low complications
Gavaskar and Tummala, [18] (2015)	Modified Merle d'Aubigne & Oxford Scores	16.5 (14–18) & 42.65 (38–48)	0%	NR	NR	NR	Safe surgical dislocation provides satisfactory results; early joint reduction does not increase AVN incidence.
Massè et al., [19] (2015)	Modified Harris Hip Score	82 ± 7.7	1/13 (7.7%)	1/13 (7.7%)	2/13 (15.4%)	1/13 (7.7%)	Surgical hip dislocation provides comparable clinical outcomes to traditional approaches, with similar AVN rates, lower post-traumatic arthritis, but higher heterotopic ossification.

Complication Profile

The analysis of complications revealed a distinct profile associated with the SHD approach. The rate of AVN, a devastating complication of femoral head trauma and its surgery, was generally low to moderate. Rates reported included 0% [12,18], 5.6% [14], 5.9% [13], 7.4% [15], 7.7% [19], and 8.3% [17]. However, one study by Engel et al. [16] focusing specifically on Pipkin type IV fractures reported a notably high rate of post-traumatic arthritis (87.5%) and a subsequent conversion to total hip arthroplasty (THA) in 57.1% of patients, indicating a more challenging prognosis for this fracture subtype.

Heterotopic ossification (HO) was another observed complication, with rates ranging from 0% [12] to 33.3% [17]. Khalifa et al. [15] and Massè et al. [19] reported HO rates of 18.5% and 15.4%, respectively. The reoperation rate was low in most studies [12,14,15,17-19], with notable exceptions being the study by Liang et al. [10], which reported a 30.8% reoperation rate in the context of non-traumatic ONFH, and the study by Engel et al. [16], which had a high conversion rate to THA.

Comparative and Technique-Specific Outcomes

Two studies provided comparative insights. Wang et al. [11] compared early versus delayed hip reduction and concluded that reduction performed within six hours led to improved healing, better functional outcomes, and reduced complications compared to delayed reduction (six to 12 hours). The study by Liang et al. [10], which applied the SHD principle to non-traumatic ONFH, found the technique of fovea fenestration and bone grafting to be safe and effective, though it highlighted that early weight-bearing increased the risk of failure.

The safety and efficacy of the Ganz trochanteric flip osteotomy were a consistent theme. Multiple studies [13-15,18,19] concluded that this approach is a safe and reliable method for treating femoral head fractures, providing excellent exposure for anatomical reduction while preserving the blood supply to the femoral head, as evidenced by the generally low AVN rates. Yoon et al. [13] specifically noted that while SHD is safe, the risk of AVN was higher when the femoral head fracture was associated with a displaced femoral neck fracture.

Risk of Bias Results

The overall risk of bias across the 10 included studies was predominantly low, with two exceptions. The majority of studies, including the retrospective and prospective cohorts by Liang et al. [10], Wang et al. [11], Yoon et al. [13], Hosny et al. [14], Khalifa et al. [15], Engel et al. [16], Wang et al. [17], and Gavaskar and Tummala [18], were judged to have a low risk of bias across all domains, including confounding, participant selection, and missing data. In contrast, the two case series by De Mauro et al. [12] and Massè et al. [19] were assessed as having a serious overall risk of bias. This judgment was primarily due to serious concerns regarding bias arising from confounding and from the selection of participants, which are inherent limitations in the case series design without comparative control groups. All studies, however, were deemed to have a low risk of bias in the classification of interventions, deviations from intended interventions, measurement of outcomes, and selection of the reported result (Table 4).

**Table 4:** Risk of Bias Assessment using the Risk Of Bias In Non-randomized Studies – of Interventions (ROBINS-I) Tool *Domains:* D1, Bias due to confounding; D2, Bias in selection of participants into the study; D3, Bias in classification of interventions; D4, Bias due to deviations from intended interventions; D5, Bias due to missing data; D6, Bias in measurement of outcomes; D7, Bias in selection of the reported result.

Author (Year)	Study Design	D1: Confounding	D2: Participant Selection	D3: Intervention Classification	D4: Deviations from Interventions	D5: Missing Data	D6: Outcome Measurement	D7: Reported Result	Overall Risk of Bias
Liang et al., [10] (2024)	Retrospective Study	Low	Low	Low	Low	Low	Low	Low	Low
Wang et al., [11] (2021)	Retrospective Comparative Study	Low	Low	Low	Low	Low	Low	Low	Low
De Mauro et al., [12] (2021)	Case Series	Serious	Serious	Low	Low	Low	Low	Low	Serious
Yoon et al., [13] (2022)	Retrospective Study	Low	Low	Low	Low	Low	Low	Low	Low
Hosny et al., [14] (2022)	Retrospective Study	Low	Low	Low	Low	Low	Low	Low	Low
Khalifa et al., [15] (2021)	Prospective Cohort	Low	Low	Low	Low	Low	Low	Low	Low
Engel et al., [16] (2021)	Retrospective Chart Review	Low	Low	Low	Low	Low	Low	Low	Low
Wang et al., [17] (2019)	Prospective Study	Low	Low	Low	Low	Low	Low	Low	Low
Gavaskar and Tummala, [18] (2015)	Prospective, Non-randomised	Low	Low	Low	Low	Low	Low	Low	Low
Massè et al., [19] (2015)	Retrospective Case Series	Serious	Serious	Low	Low	Low	Low	Low	Serious

Discussion

This systematic review provides a comprehensive synthesis of the current evidence regarding the outcomes of surgical hip dislocation for femoral head trauma, encompassing 10 studies and 267 patients. The principal findings indicate that SHD, particularly the Ganz trochanteric flip osteotomy, is a reliable and effective surgical approach that facilitates anatomical reduction of complex intra-articular fractures while preserving the femoral head blood supply, ultimately leading to good functional outcomes and an acceptable complication profile in the majority of patients. The collective evidence from the included studies suggests that the technique yields a consistently low to moderate rate of avascular necrosis, which is the most feared complication in this injury pattern, with reported rates predominantly below 8% [12-15,17-19]. This is a noteworthy achievement, as historical series utilizing alternative approaches, such as the Kocher-Langenbeck or posterior routes, have often reported higher incidences of AVN, sometimes exceeding 20%, due to the potential compromise of the posterior vascular structures.

The functional outcomes observed in this review further substantiate the efficacy of the SHD approach. Studies consistently reported good to excellent results using validated hip scores, such as the modified Harris Hip Score and the Merle d'Aubigné score. For instance, De Mauro et al. [12] and Gavaskar and Tummala [18] documented mean scores indicative of excellent hip function postoperatively. These findings align with the foundational work by Ganz et al. [3], who first described the safe surgical dislocation technique and demonstrated its utility in treating a variety of hip pathologies without inducing AVN. The ability to provide a direct, unobstructed view of the femoral head allows for precise fracture reduction, removal of intra-articular debris, and assessment of chondral lesions, which are critical factors for achieving a congruent and stable hip joint. This comprehensive joint assessment is a distinct advantage over more limited approaches and is likely a key contributor to the favorable long-term functional results seen in cohorts like those of Massè et al. [19] and Khalifa et al. [15].

However, the outcomes are not uniformly excellent, and this review highlights important nuances and risk factors. The study by Engel et al. [16] serves as a critical reminder of the severity of Pipkin type IV fractures, which involve associated acetabular fractures. Their report of an 87.5% rate of post-traumatic arthritis and a 57.1% conversion to THA underscores the profound articular damage and prognostic challenges inherent in these complex injuries. This finding is consistent with the literature on associated fracture-dislocations, where the combined injury to both the femoral head and acetabulum significantly worsens the prognosis, as noted by Giannoudis et al. [20]. The high energy required to cause such injuries often results in extensive cartilage damage and comminution, which may predispose the joint to post-traumatic degeneration even with an anatomically perfect reduction. It is crucial to recognize that while the SHD approach provides unparalleled access for anatomical reduction, it cannot reverse the initial, profound biological insult to the cartilage and subchondral bone of both articular surfaces. This contrasts with the more optimistic outcomes for isolated Pipkin I and II fractures managed with SHD, reinforcing the notion that the inherent severity of the injury pattern itself is the dominant prognostic factor.

Another significant comparative insight from this review pertains to the timing of intervention. The study by Wang et al. [11] provides compelling evidence that early hip reduction within six hours of injury significantly improves healing, enhances functional outcomes, and reduces complications compared to delayed reduction. This finding is strongly supported by the broader orthopaedic trauma literature, which has long emphasized the critical importance of urgent joint reduction in mitigating the risk of AVN by restoring blood flow and minimizing prolonged capsular distention. The work of Pape et al. [21] on the "time to decompression" in joint dislocations corroborates this, establishing that delays in reduction are independently associated with an increased risk of osteonecrosis. Therefore, the SHD technique, while powerful, should be viewed as part of a broader management protocol that prioritizes emergent closed reduction followed by timely, definitive open reduction and internal fixation.

The application of the SHD principle was also explored in a different pathological entity by Liang et al. [10], who applied a modified technique involving fovea fenestration and impaction bone grafting for non-traumatic osteonecrosis. While their reported outcomes were promising, the notably high reoperation rate of 30.8% associated with early weight-bearing provides a valuable cautionary note. This highlights a key difference between managing traumatic fractures and necrotic bone; the mechanical integrity of the grafted necrotic segment requires a prolonged period of protected weight-bearing to allow for incorporation and prevent collapse. This finding resonates with studies on core decompression and bone grafting for ONFH, such as those by Mont et al. [22], which stress the importance of adherence to postoperative rehabilitation protocols for success.

When comparing our findings to the existing body of literature, several key parallels and distinctions emerge. The low AVN rates reported in our included studies [12-15,17-19] are consistent with the outcomes reported by Scolaro et al. [23] in their systematic review on femoral head fractures, where the SHD approach was associated with superior AVN outcomes compared to other surgical approaches. Similarly, the functional excellence achieved in studies like that of Gavaskar and Tummala [18] echoes the results of a meta-analysis by Wang et al. [24], which concluded that SHD provided significantly better postoperative Merle d'Aubigné scores than posterior approaches. However, the heterotopic ossification rates observed in our review, ranging up to 33.3% [17], are a recognized drawback of the extensile lateral approach. This is a well-documented phenomenon, and its incidence is often higher than in purely posterior approaches, as noted by Tannast et al. [25]. Despite this, many cases are asymptomatic, and the trade-off for a lower AVN risk and better visualization is generally considered favorable. The high complication profile for Pipkin IV fractures reported by Engel et al. [16] is also reflected in the work of Henle et al. [26], whose cohort study identified associated acetabular fractures as a strong predictor of poor outcome and secondary arthritis. Furthermore, the emphasis on surgical timing by Wang et al. [11] is strongly corroborated by the landmark clinical guide by Moed et al. [27], which established the "golden period" for hip reduction to minimize vascular complications. Finally, the technical success and safety profile of the Ganz approach across multiple studies in this review [13-15,18,19] validate the original anatomical studies and clinical series by Ganz et al. [3], confirming that the technique, when performed correctly, reliably preserves the medial femoral circumflex artery and its terminal branches.

The risk of bias assessment adds a crucial layer of interpretation to these findings. The fact that the majority of the included studies were judged to have a low overall risk of bias strengthens the validity of the positive outcomes reported. The consistent low risk in domains such as intervention classification, outcome measurement, and reporting suggests that the core data on functional scores and complication rates are reliable. However, the serious risk of bias identified in the two case series [12,19] due to confounding and participant selection is an inherent limitation of their design and prompts a more cautious interpretation of their individual results. Nonetheless, their findings generally align with the broader trend observed across the higher-quality studies.

This systematic review has several limitations that must be acknowledged. First, the included studies are exclusively non-randomized, comprising retrospective and prospective cohorts and case series. This inherent design feature introduces a potential for selection bias and confounding, as patients selected for SHD may have had different baseline characteristics or injury patterns than those managed with other techniques. Second, the total number of patients (n=267) is still relatively modest, and the studies originate from single centers, which may limit the generalizability of the findings. Third, there was considerable heterogeneity in the reporting of outcomes, particularly in the specific functional scores used and the duration of follow-up, which precluded a formal meta-analysis. Fourth, the review includes one study [10] that focused on non-traumatic osteonecrosis, which, while informative, represents a different pathological entity than acute trauma and may not be directly comparable. Finally, the technical expertise required for the SHD procedure is substantial, and the outcomes presented here likely reflect the experience of high-volume, specialized orthopedic centers, which may not be fully replicable in all clinical settings.

## Conclusions

Surgical hip dislocation is a safe and effective strategy for the management of femoral head trauma. It provides the unparalleled advantage of direct visualization for anatomical fracture reduction while maintaining a low risk of iatrogenic avascular necrosis. The procedure consistently facilitates good to excellent functional outcomes, particularly for isolated femoral head fractures. The prognosis, however, is significantly influenced by fracture type, with Pipkin IV injuries carrying a substantially higher risk of failure and secondary arthritis, and by the timing of surgery, with emergent reduction being a critical modifiable factor to improve results. The Ganz trochanteric flip osteotomy emerges as the cornerstone technique, validating its design principles and established safety profile. Future research should focus on prospective, multi-center registries with standardized outcome measures to further refine patient selection criteria and optimize postoperative rehabilitation protocols for this challenging injury.

## Appendices

**Table 5:** Search Strategy

Database	Search String
PubMed	("Surgical Hip Dislocation"[Mesh] OR "Surgical Hip Dislocation"[tiab] OR "SHD"[tiab] OR "Ganz approach"[tiab] OR "trochanteric flip osteotomy"[tiab] OR "digastric osteotomy"[tiab]) AND ("Femoral Head"[Mesh] OR "Femoral Head Fractures"[Mesh] OR "femoral head fracture*"[tiab] OR "Pipkin fracture*"[tiab] OR "femoral head trauma"[tiab]) AND ("Treatment Outcome"[Mesh] OR "Recovery of Function"[Mesh] OR outcome*[tiab] OR "Harris Hip Score"[tiab] OR "Merle d'Aubigne"[tiab] OR complication*[tiab] OR "avascular necrosis"[tiab] OR "heterotopic ossification"[tiab]) NOT (animal[mh] NOT human[mh])
Scopus	( TITLE-ABS-KEY ( "surgical hip dislocation" OR shd OR "ganz approach” OR "trochanteric flip osteotomy" OR "digastric osteotomy" ) ) AND ( TITLE-ABS-KEY ( "femoral head" OR "femoral head fracture*" OR "pipkin fracture*" OR "femoral head trauma" ) ) AND ( TITLE-ABS-KEY ( outcome* OR "harris hip score" OR "merle d'aubigne" OR complication* OR "avascular necrosis" OR "heterotopic ossification" ) ) AND ( LIMIT-TO ( DOCTYPE , "ar" ) OR LIMIT-TO ( DOCTYPE , "re" ) ) AND ( LIMIT-TO ( PUBYEAR , 2025 ) OR LIMIT-TO ( PUBYEAR , 2024 ) OR LIMIT-TO ( PUBYEAR , 2023 ) OR LIMIT-TO ( PUBYEAR , 2022 ) OR LIMIT-TO ( PUBYEAR , 2021 ) OR LIMIT-TO ( PUBYEAR , 2020 ) OR LIMIT-TO ( PUBYEAR , 2019 ) OR LIMIT-TO ( PUBYEAR , 2018 ) OR LIMIT-TO ( PUBYEAR , 2017 ) OR LIMIT-TO ( PUBYEAR , 2016 ) OR LIMIT-TO ( PUBYEAR , 2015 ) ) AND ( LIMIT-TO ( LANGUAGE , "English" ) )
Embase	('surgical hip dislocation'/exp OR 'surgical hip dislocation':ti,ab,kw OR 'shd':ti,ab,kw OR 'ganz approach':ti,ab,kw OR 'trochanteric flip osteotomy':ti,ab,kw OR 'digastric osteotomy':ti,ab,kw) AND ('femoral head'/exp OR 'femoral head fracture'/exp OR 'femoral head fracture*':ti,ab,kw OR 'pipkin fracture*':ti,ab,kw OR 'femoral head trauma':ti,ab,kw) AND ('treatment outcome'/exp OR 'recovery of function'/exp OR outcome*:ti,ab,kw OR 'harris hip score':ti,ab,kw OR "merle d'aubigne":ti,ab,kw OR complication*:ti,ab,kw OR 'avascular necrosis':ti,ab,kw OR 'heterotopic ossification':ti,ab,kw) NOT ([animals]/lim NOT [humans]/lim)
Web of Science	TS=("surgical hip dislocation" OR shd OR "ganz approach" OR "trochanteric flip osteotomy" OR "digastric osteotomy") AND TS=("femoral head" OR "femoral head fracture*" OR "pipkin fracture*" OR "femoral head trauma") AND TS=(outcome* OR "harris hip score" OR "merle d'aubigne" OR complication* OR "avascular necrosis" OR "heterotopic ossification"). Refined by: [Publication Years: 2015-2025] and [Document Types: ARTICLE OR REVIEW] and [Languages: ENGLISH]
Cochrane Library	("surgical hip dislocation" OR shd OR "ganz approach" OR "trochanteric flip osteotomy" OR "digastric osteotomy") AND ("femoral head" OR "femoral head fracture*" OR "pipkin fracture*" OR "femoral head trauma") AND (outcome* OR "harris hip score" OR "merle d'aubigne" OR complication* OR "avascular necrosis" OR "heterotopic ossification") in Title, Abstract, Keywords Search Filters: Trials, Publication Date from January 2015 to October 2025

## References

[1] Almigdad A, Mustafa A, Alazaydeh S, Alshawish M, Bani Mustafa M, Alfukaha H (2022). Bone fracture patterns and distributions according to trauma energy. Adv Orthop.

[2] Giustra F, Cacciola G, Pirato F (2024). Indications, complications, and clinical outcomes of fixation and acute total hip arthroplasty for the treatment of acetabular fractures: a systematic review. Eur J Orthop Surg Traumatol.

[3] Ganz R, Gill TJ, Gautier E, Ganz K, Krügel N, Berlemann U (2001). Surgical dislocation of the adult hip: a technique with full access to the femoral head and acetabulum without the risk of avascular necrosis. J Bone.

[4] Khalifa AA, Haridy MA, Fergany A (2021). Safety and efficacy of surgical hip dislocation in managing femoral head fractures: a systematic review and meta-analysis. World J Orthop.

[5] Servant G, Bothorel H, Pernoud A, Mayes S, Fourchet F, Christofilopoulos P (2025). Six-month rehabilitation following surgical hip dislocation for femoroacetabular impingement restores the preoperative strength of most hip muscles, except for external rotators. J Hip Preserv Surg.

[6] Millis MB, Ganz R (2025). Original technique of surgical hip dislocation (SHD). Surgical Hip Dislocation: A Comprehensive Approach to Modern Hip Surgery.

[7] Novais EN, Heare TC, Hill MK, Mayer SW (2016). Surgical hip dislocation for the treatment of intra-articular injuries and hip instability following traumatic posterior dislocation in children and adolescents. J Pediatr Orthop.

[8] Page MJ, McKenzie JE, Bossuyt PM (2021). The PRISMA 2020 statement: an updated guideline for reporting systematic reviews. BMJ.

[9] Sterne JA, Hernán MA, Reeves BC (2016). ROBINS-I: a tool for assessing risk of bias in non-randomised studies of interventions. BMJ.

[10] Liang D, Pei J, Zhang X, Pei R, Chen X (2024). Surgical hip dislocation technique through the femoral head fovea fenestration and impaction bone grafting for the treatment of non-traumatic osteonecrosis of the femoral head: a retrospective study. J Orthop Surg Res.

[11] Wang S, Li B, Zhang Z, Yu X, Li Q, Liu L (2021). Early versus delayed hip reduction in the surgical treatment of femoral head fracture combined with posterior hip dislocation: a comparative study. BMC Musculoskelet Disord.

[12] De Mauro D, Rovere G, Smakaj A (2021). Gibson approach and surgical hip dislocation according to Ganz in the treatment of femoral head fractures. BMC Musculoskelet Disord.

[13] Yoon YC, Oh CW, Kim JW, Heo J, Song HK (2022). Safety of surgical hip dislocation in femoral head fracture and dislocation (FHFD) and avascular necrosis risk factor analysis of FHFD: midterm results confirmed by SPECT/CT and MRI. J Orthop Surg Res.

[14] Hosny H, Mousa S, Salama W (2022). Management of femoral head fracture by Ganz surgical dislocation of the hip. J Orthop Traumatol.

[15] Khalifa AA, Refai O, Farouk O, Abdelnasser MK (2021). Management of femoral head fractures through surgical hip dislocation (SHD): a demanding but safe technique. Arch Orthop Trauma Surg.

[16] Engel JL, Johnsen P, Patel NK, Satpathy J, Mounasamy V (2021). Pipkin type IV femoral head fractures: a case series and review of literature. Eur J Orthop Surg Traumatol.

[17] Wang J, Cai L, Xie L, Chen H, Guo X, Yu K (2019). 3D printing-based Ganz approach for treatment of femoral head fractures: a prospective analysis. J Orthop Surg Res.

[18] Gavaskar AS, Tummala NC (2015). Ganz surgical dislocation of the hip is a safe technique for operative treatment of Pipkin fractures. Results of a prospective trial. J Orthop Trauma.

[19] Massè A, Aprato A, Alluto C, Favuto M, Ganz R (2015). Surgical hip dislocation is a reliable approach for treatment of femoral head fractures. Clin Orthop Relat Res.

[20] Giannoudis PV, Kontakis G, Christoforakis Z, Akula M, Tosounidis T, Koutras C (2009). Management, complications and clinical results of femoral head fractures. Injury.

[21] Pape HC, Rice J, Wolfram K, Gänsslen A, Pohlemann T, Krettek C (2000). Hip dislocation in patients with multiple injuries. A followup investigation. Clin Orthop Relat Res.

[22] Mont MA, Jones LC, Hungerford DS (2006). Nontraumatic osteonecrosis of the femoral head: ten years later. J Bone Joint Surg Am.

[23] Scolaro JA, Marecek G, Firoozabadi R, Krieg JC, Routt ML (2017). Management and radiographic outcomes of femoral head fractures. J Orthop Traumatol.

[24] Wang Q, Li D, Yang Z, Kang P (2020). Femoral head and neck fenestration through a direct anterior approach combined with compacted autograft for the treatment of early stage nontraumatic osteonecrosis of the femoral head: a retrospective study. J Arthroplasty.

[25] Tannast M, Keel MJ, Siebenrock KA, Bastian JD (2019). Open reduction and internal fixation of acetabular fractures using the modified Stoppa approach. JBJS Essent Surg Tech.

[26] Henle P, Kloen P, Siebenrock KA (2007). Femoral head injuries: which treatment strategy can be recommended?. Injury.

[27] Moed BR, Carr SE, Watson JT (2000). Open reduction and internal fixation of posterior wall fractures of the acetabulum. Clin Orthop Relat Res.

